# Toward Identifying a Multivariate Correlation of Septic Arthritis With a Machine Learning Approach: Time to Reset the Current Australasian Guidelines?

**DOI:** 10.1111/1756-185x.70386

**Published:** 2025-08-15

**Authors:** Sourav Bhattacharjee, Imal C. Hemachandra, Sudharsan Venkatesan, Robert W. Baird, Sachin Khetan

**Affiliations:** ^1^ School of Veterinary Medicine University College Dublin Dublin 4 Ireland; ^2^ Conway Institute of Biomolecular and Biomedical Research University College Dublin Dublin 4 Ireland; ^3^ Department of Infectious Diseases Royal Darwin Hospital Casuarina Northern Territory Australia; ^4^ Territory Pathology Royal Darwin Hospital Casuarina Northern Territory Australia; ^5^ Department of General Medicine Royal Darwin Hospital Tiwi Northern Territory Australia

**Keywords:** aseptic arthritis, data mining, logistic regression, multivariate correlation of disease, ROC analysis, sensitivity, septic arthritis, specificity, synovial leucocyte count

## Abstract

**Objectives:**

To understand the complexity of disease pathology through the prism of septic arthritis, especially the reliability of popular and, yet, arbitrary thresholds like synovial leucocyte counts of ≥ 100,000/μL suggestive of it, with the help of statistical analysis and logistic regression.

**Methods:**

An anonymized patient dataset comprising 360 swollen joint episodes was collated along with a range of patient attributes, including age, gender, comorbidity (e.g., diabetes, gout, pseudogout, immunosuppression), prior administration of antibiotics and washout of the affected joint, isolation of crystals from synovial aspirate, blood/synovial fluid culture growth, and synovial aspirate cell count. The dataset was subjected to statistical analysis (e.g., sensitivity, specificity, predictive and likelihood ratios) and logistic regression modeling, with results compared to the synovial leucocyte count thresholds of ≥ 100,000/μL and ≥ 50,000/μL.

**Results:**

The logistic regression model (sensitivity 50%, specificity 97.04%) outperformed the models based on arbitrary thresholds like a synovial leucocyte count of ≥ 100,000/μL (sensitivity 48.21%, specificity 88.16%) or ≥ 50,000/μL (sensitivity 64.29%, specificity 69.74%) in predicting septic arthritis. Independent variables like age, presence of gout, and autoimmune arthritis as comorbidities, hip joint involvement, synovial aspirate leucocyte count, and crystals in aspirated fluid demonstrated a significant (*p* < 0.05) correlation to septic arthritis.

**Conclusion:**

Septic arthritis presents a multivariate correlation that deserves a holistic oversight rather than singling out individual factors. Data mining platforms like logistic regression can investigate the complex interplay among these individual variables while making a diagnosis not only in septic arthritis but also in other diseases with multisystem involvement, infective or non‐infective alike.

## 1 Introduction

The association of septic arthritis (SA) with rheumatoid arthritis (RA), especially in RA patients on anti‐tumor necrosis factor‐alpha therapy, is well established and is often considered a hallmark of worse prognosis [1]. Joint sepsis is frequently caused by bacterial infection (80%) [2], although viruses [3], mycobacteria [4], and fungi [5] may also be responsible. Other than *Neisseria gonorrhoeae* , which causes gonococcal arthritis in young sexually active adults [6], *Staphylococcus aureus* (including Methicillin‐Resistant *Staphylococcus Aureus* (MRSA) strains, 40%), *Streptococcus pneumoniae* (28%), *Pseudomonas aeruginosa* , *Haemophilus influenzae* , and *Escherichia coli* are frequently isolated from the affected joints in non‐gonococcal arthritis [3]. The association between SA and RA is attributed to a variety of factors, including immunosuppression (often due to prolonged steroid therapy) [7], damaged articular surfaces with synovial neovascularization [8], and impaired phagocytosis by the polymorphonuclear leucocytes in the synovial fluid [9].

Other than RA, underlying risk factors of SA include diabetes [10], osteoarthritis [11], immunosuppressed states due to senility [12], prior medication or HIV infection [13], recent joint replacement [14], gout [15], and a history of intravenous drug abuse [16]. The incidence of SA varies between 4 and 29 per 100,000 person‐years, while 85% of patients reported the contributing factors mentioned earlier [17]. In Australia, the incidence of SA at the Top End of the Northern Territory between 1978 and 1994 was 9.2 per 100,000 person‐years and 29 per 100,000 person‐years among Aboriginal Australians [18]. Diabetes, corticosteroid administration, pre‐existing joint diseases, and alcohol abuse were identified among the commonest risk factors. Non‐gonococcal SA accounted for 88% of cases, while 80%–90% were monoarticular episodes.

An early diagnosis remains crucial for the effective management of SA with limited disability and morbidity. Due to a lack of clinical correlation and inadequate symptomatic findings, arthrocentesis remains the mainstay, while synovial aspirate bacterial culture is the gold standard of SA diagnosis [19]. Unfortunately, a global consensus on the synovial leucocyte count threshold to segregate the SA cases from aseptic arthritis (AA) is missing, although the current Royal College of Pathologists Australasia (2019) guidelines, followed extensively in Australia and New Zealand, use synovial leucocyte counts of ≥ 100 000/μL and ≥ 50,000/μL as diagnostic markers of SA and bacterial arthritis, respectively [20].

However, using such synovial leucocyte count thresholds to separate the SA cohort from AA remains controversial. Drawing such arbitrary watershed lines between SA and AA also ignores the role of contributing factors. The way a *disease* is defined in modern medicine is undergoing a major overhaul, and a paradigm shift is emerging where, instead of a univariate model, a multivariate analysis of disease etiology is gaining traction [21]. The core concept here encompasses an idea that the disease is a collective expression, clinically ingrained as the *signs* and *symptoms*, of a broader, interrelated web of factors that, in cohesion, cause the pathology as an outcome. Thus, other factors must be weighed to make a clinical diagnosis methodologically robust, clinically justified, and rationally defensible.

Finally, synovial leucocyte count and culture vary based on other factors, such as prior antibiotic therapy, a common scenario in SA that commences before joint aspiration and invariably influences the synovial leucocyte count [22]. Similarly, peripheral blood (sensitivity 26%–36%) or synovial fluid (sensitivity 29%–65%) culture, including Gram staining, has produced weaker correlation patterns that, apart from being statistically insignificant, also suffered from other inadequacies. Despite being a helpful research tool, the Newman classification [23, 24, 25], which linked isolated microorganisms from the affected joint or elsewhere in the patient's body to corroborate SA, also suffers from erratic correlation.

The aims and objectives of the study were: (i) to collect and curate a retrospective (anonymized) dataset of patients with arthritis; (ii) to explain the dataset and understand the extent of SA or AA with descriptive statistical parameters; (iii) to analyze the dataset and develop a binary (SA or AA) modeling paradigm with logistic regression; (iv) to comprehend how the independent variables, including synovial leucocyte count and allied comorbidities, influenced the final diagnosis (dependent variable) of SA or AA; (v) to compare the obtained results with the ones derived from synovial leucocyte count‐based thresholds of 100 000/μL and 50 000/μL on the merit of performance and discrimination capability.

## 2 Patients and Methods

### 2.1 Ethical Approval

Ethics statement was obtained from the Human Research Ethics Committee of the Northern Territory Department of Health and Menzies School of Health Research to conduct an observational retrospective cross‐sectional study for patients managed in public hospitals within the Top End Health Service (Royal Darwin Hospital, Katherine District Hospital, and Gove District Hospital), with an approval code: EFILE2020/25864—HREC 20–3766.

### 2.2 Patient Selection and Data Collection

Data were extracted from the local Labtrak pathology database for synovial fluid leucocyte counts of affected joints from 01/01/2017–31/12/2019 (three years). Episodes were excluded if it occurred outside the study period, had inaccessible medical records, involved extra‐articular samples, duplicate samples from the same joint, or unavailable crystal microscopy or culture results. Two researchers reviewed the Electronic Medical Record and supplemented it with paper charts, as required for the inpatient stay and correspondence from relevant clinics within three months of the index hospital discharge.

Diagnoses were defined based on what was documented in the hospital discharge summaries or outpatient clinic correspondence. Modified Newman criteria were used to classify SA cases into three tiers: definite (Newman A), probable (Newman B), and possible (Newman C). Definite SA indicated that a clinically significant microorganism was isolated from the joint and identified by Gram staining, culture, or polymerase chain reaction. Probable SA was defined as a significant microorganism isolated in a clinical specimen collected from elsewhere other than the affected joint. Finally, a possible SA had no microorganism isolated, but physicians made the diagnosis based on available information, including (but not limited to) clinical examination, synovial fluid cell counts, radiology, histology, and response to antibiotics. The length of stay included hospital‐in‐the‐home and inpatient rehabilitation. Immunosuppression was defined as per the hospital guidelines. The duration of antibiotic administration included treating coexisting infections, such as endocarditis or osteomyelitis.

The collated data included information on (anonymous) patient ID, gender, age, presence of immunosuppression, diabetes, gout, and pseudogout, autoimmune arthritis (RA and seronegative arthritis), Charlson comorbidity index (CCI) [26], any history of prior administration of antibiotics (and if yes then duration in days), Newman score (A, B, and C), length of hospital stay in days, synovial aspirate cell count, history of joint washout(s), any microbial growth noticed in blood or synovial fluid culture, presence of crystals in synovial fluid, and diagnosis (as mentioned in the discharge summary). The attributes were further classified as metric (age, antimicrobial duration, aspirate cell count/μL, and hospital stay), ordinal (CCI), and nominal (patient ID, gender, immunosuppression, diabetes, gout, pseudogout, autoimmune arthritis, antibiotic, joint aspirated, single joint, washout, synovial culture, blood culture, crystals, limb (upper/lower), (if) inflammatory, and diagnosis). The assimilated data were tabulated in an MS Excel spreadsheet (Microsoft Corporation, Redmond, WA, USA). A collinearity check between the independent variables was not feasible, as many of them were ordinal or nominal data; therefore, a correlation matrix could not be generated. However, such collinearity analysis is advisable in future studies when feasible.

### 2.3 Data Analysis

The descriptive statistical analysis and logistic regression modeling were conducted online at DATAtab (https://datatab.net/), a web‐based statistical application with a live calculation tracker. The MS Excel spreadsheet was uploaded into its interface using the *Export/Import* tab, followed by designating the columns as nominal, ordinal, or metric variables. The *Descriptive Statistics* wizard was then used to calculate the location (mean and median) and dispersion (standard deviation, variance, and range) parameters.

### 2.4 Logistic Regression Modeling

A logistic regression analysis was conducted as an *Inferential Statistics* exercise where the logistic function $f\left(z\right)$ was defined as:

$$f\left(z\right)=\frac{1}{1+e^{-z}}$$

$$\Rightarrow z=b_{1}\cdot x_{1}+b_{2}\cdot x_{2}+b_{3}\cdot x_{3}+\ldots +b_{k-1}\cdot x_{k-1}+b_{k}\cdot x_{k}+b$$

Here, $x_{1}$, $x_{2}$, $x_{3}$, …, $x_{k-1}$, $x_{k}$ were independent variables, whereas, $b_{1}$, $b_{2}$, $b_{3}$, …, $b_{k-1}$, $b_{k}$ and b stood for the regression coefficients and regression constant, respectively.

For binary logistic regression analysis (SA or AA), the same online DATAtab online platform was used, while the reference categories for the attributes were as follows: gender–female, immunosuppression–absent, diabetes–absent, gout–absent, pseudogout–absent, autoimmune arthritis–absent, joint aspirated–elbow, single joint–yes, CCI–0, and crystals–no. Data on prior joint washout and inflammation were excluded as confounding factors. The duration of antibiotic therapy was excluded from the predictive model due to the retrospective nature of this study, while its selection would have skewed the predictive ability of the model (by pre‐selecting patients who were treated for SA already).

The Chi‐square (*χ* ^2^) value was assessed, with a statistical significance level set at *p* < 0.05. Furthermore, the sensitivity and specificity were determined. The null hypothesis was that there was no relationship between the observed and expected frequencies of the outcomes predicted by the model. The goodness of fit was measured by −2 Log‐Likelihood and Cox & Snell *R* ^2^ [27], Nagelkerke *R* ^2^ [28], and McFadden's *R* ^2^ [29] values. A receiver operating characteristic (ROC) curve [30] was developed based on logistic regression and synovial leucocyte‐based thresholds of ≥ 100,000/μL and ≥ 50,000/μL, with estimations of the area under the curve (AUC) for all models.

### 2.5 Statistical Analysis

Using a confusion matrix for logistical regression modeling, the following statistical parameters were measured: sensitivity, specificity, positive and negative predictive values, positive and negative likelihood ratios, prevalence, and accuracy. For comparison purposes, similar confusion matrices were created for the commonly used synovial leucocyte count thresholds of ≥ 100,000/μL and ≥ 50,000/μL, while identical statistical parameters were calculated. Data plotting was done with OriginPro 2024b software (OriginLab Corporation, Northampton, MA, USA).

## 3 Results

### 3.1 Patient Demographics and Descriptive Statistics

**FIGURE 1:**
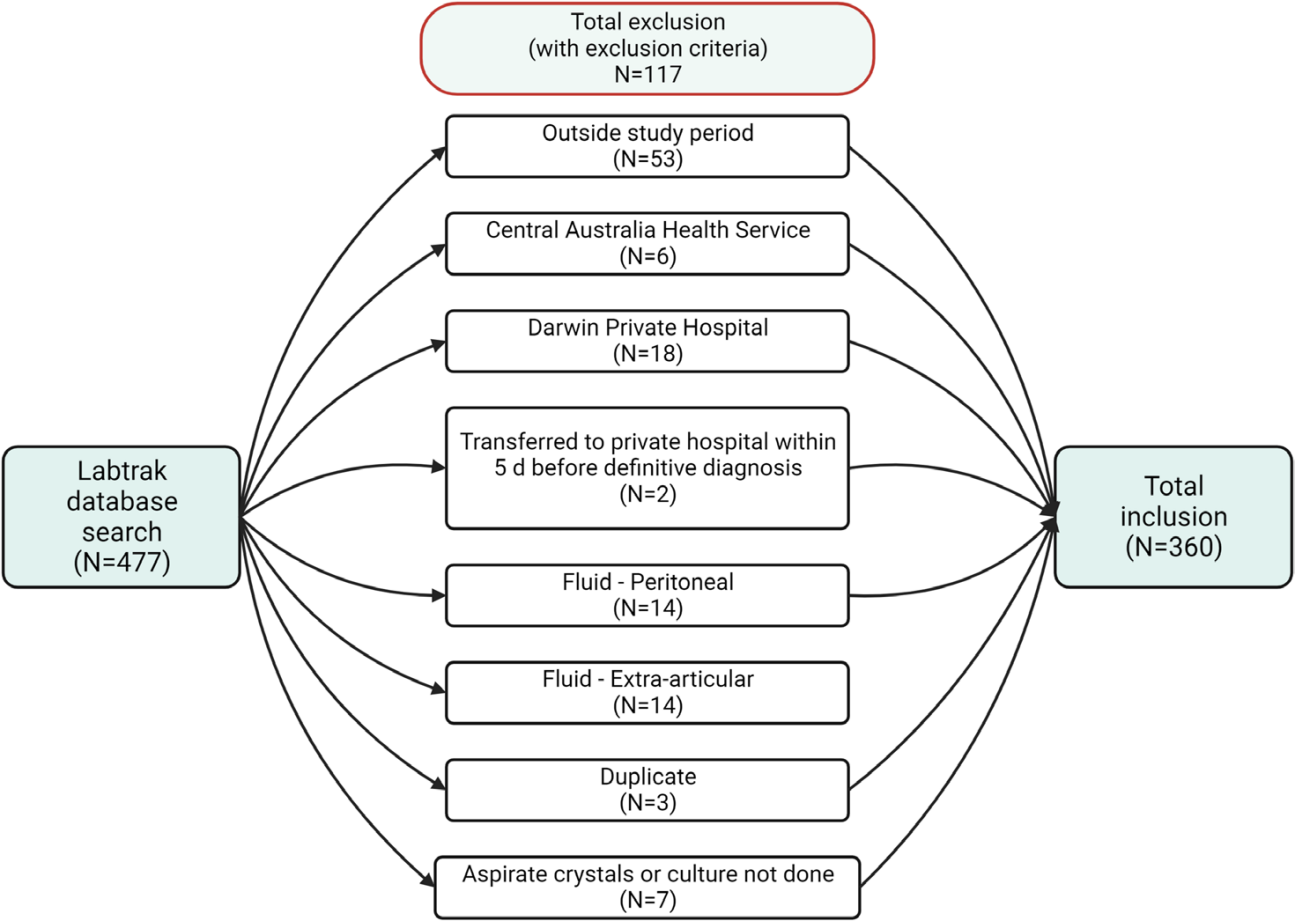
Scheme showing the methodology followed, including the exclusion criteria, to build the dataset. The number of included or excluded episodes is shown as counts (*N*).

A cohort of 477 aspirated joints was identified from the Labtrak database, and 117 were excluded based on the exclusion criteria, resulting in a final dataset comprising 360 episodes (Figure 1). Within the dataset, 15.56% (56/360) were diagnosed as SA and 84.44% (304/360) as AA. The mean age of the entire cohort was 50.57 years (range 0–90 years, standard deviation/SD 18.98 years). The cohort consisted of 71.11% males (range 90 years, mean age 51.52 years, SD 17.09 years) and 28.89% females (range 84 years, mean age 48.23 years, SD 19.91 years). Patients with SA were younger (mean age 44.13 years) than the AA (mean age 51.76 years) cohort. Among the 56 SA episodes, 15 were categorized as Newman A, 15 as Newman B, and 26 as Newman C. The commonest microorganism identified in SA episodes was methicillin‐sensitive *Staphylococcus aureus* (9/56), followed by MRSA strain (2/56), *Neisseria gonorrhoeae* (3/56), and (Group A) *Streptococcus pyogenes* (3/56). Other microorganisms detected included *Neisseria gonorrhoeae* , *Burkholderia pseudomallei* , *Escherichia coli* , *Streptococcus* sp. (Groups C and G), and *Kingella kingae* . Key patient demographics, comorbidity, and clinical data are summarized in Table 1, while the entire dataset is provided as Supporting Information S1.

**TABLE 1:** Summary of patient demographics of the studied dataset (*N* = 360). Where applicable, the % contributions to the representative cohort are also marked.

	Entire cohort		Only SA		Only AA	
	# Episodes	%	# Episodes	%	# Episodes	%
Total	360	100	56	100	304	100
Male	256	71.11	34	60.71	222	73.03
Female	104	28.89	22	39.29	82	26.97
Age (years)						
Mean ± SD	50.57 ± 17.98		44.13 ± 23.31		51.76 ± 16.6	
Comorbidities						
Immunosuppression	30	8.33	3	5.36	27	8.88
Diabetes	87	24.17	16	28.57	71	23.36
Gout	106	29.44	3	5.36	103	33.88
Pseudogout	7	1.94	1	1.79	6	1.97
Autoimmune arthritis	43	11.94	2	3.57	41	13.49
Joint aspirated						
Knee	290	80.56	39	69.64	251	82.57
Ankle	29	8.06	2	3.57	27	8.88
Elbow	17	4.72	2	3.57	15	4.93
Hip	10	2.78	5	8.93	5	1.64
Shoulder	9	2.5	7	12.5	2	0.66
Wrist	5	1.39	1	1.79	4	1.32
Relevant findings						
Crystals	166	46.11	9	16.07	157	51.64
Charlson Comorbidity Index						
Mean ± SD	2.04 ± 2.26		2.18 ± 2.5		2.01 ± 2.22	

Abbreviations: AA, aseptic arthritis; SA, septic arthritis; SD, standard deviation.

The mean synovial leucocyte count of the cohort was 60,578/μL (range 827 ‐ 987/μL, SD 100785). Crystals were detected in 46% of cases, and synovial cultures were positive in 3% of patients. When collected, blood cultures were positive in 11% of patients (18/159). The SA cases (Newman A, B, and C) had a similar distribution of leucocyte counts, while the mean leucocyte count in SA (range 827 ‐ 950/μL, mean leucocyte count 141 642/μL, SD 177523/μL) was higher than in AA (range 678 ‐ 987/μL, mean leucocyte count 45 645/μL, SD 69831/μL). The mean CCI of the entire cohort was 2.04 (range 11, SD 2.26). Relevant comorbidities included crystal‐induced arthritis (32%), diabetes (24%), autoimmune arthritis (12%), or immunosuppression (8%). Knees were the predominant joint aspirated (80.56%), while 81% were monoarticular episodes.

### 3.2 Effect of Pre‐Existing Comorbid Conditions on Synovial Leucocyte Count

**FIGURE 2:**
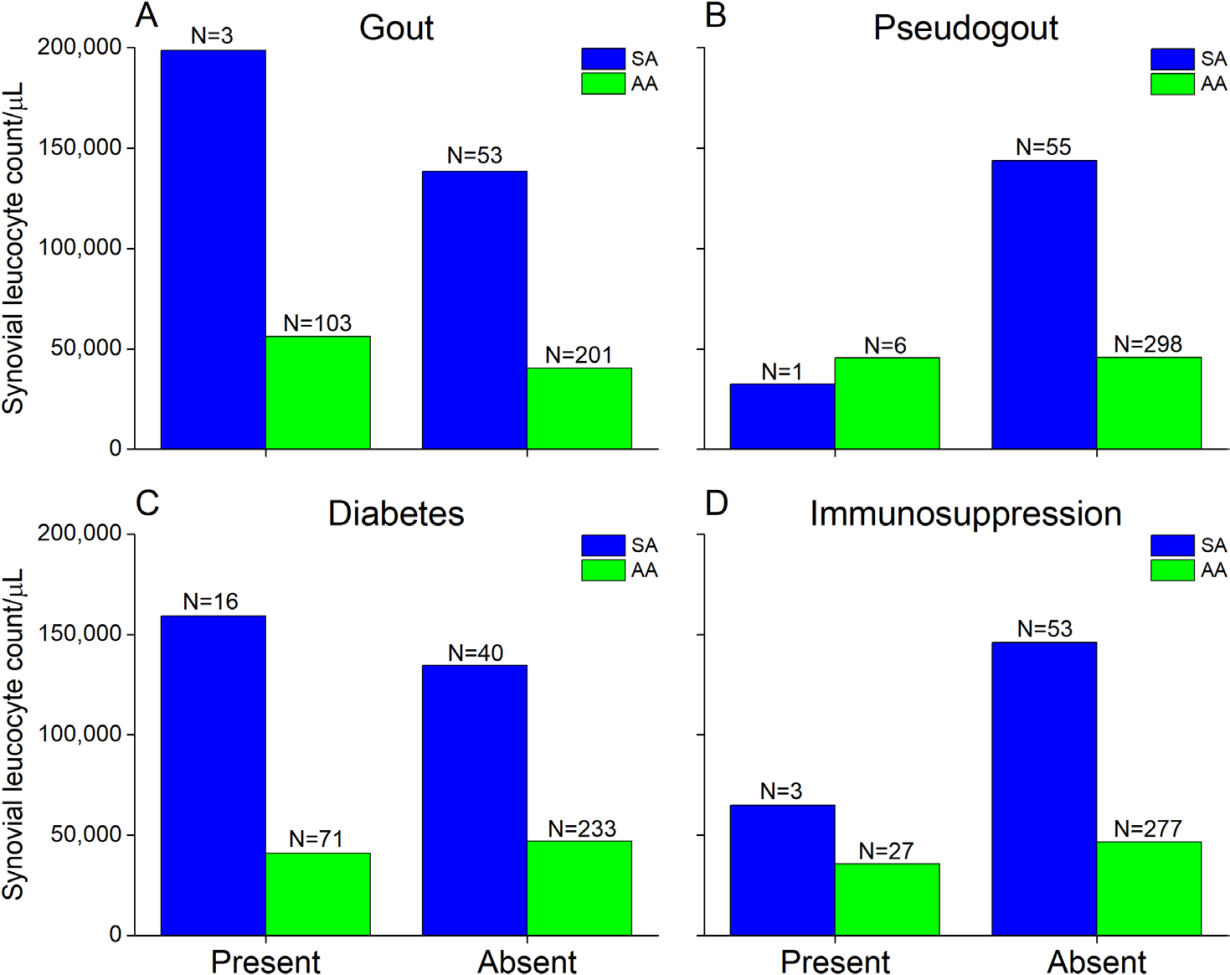
The mean synovial leucocyte counts in patients with comorbidities (A) gout; (B) pseudogout; (C) diabetes; and (D) immunosuppression. The results were further filtered along the lines of discharge diagnosis (SA and AA) while the respective counts (*N*) are mentioned.

The mean synovial leucocyte counts for comorbid conditions (gout, pseudogout, diabetes, and immunosuppression) are shown in Figure 2. The results were filtered for each comorbid condition based on the final diagnosis: SA and AA. In the case of gout (Figure 2A), the mean synovial leucocyte counts for SA and AA were 198,600/μL and 56,129/μL, respectively. Interestingly, these values were diminished for the SA (138,418/μL) and AA (40,272/μL) without gout. On the contrary, the mean synovial leucocyte counts were higher in the absence of pseudogout (Figure 2B) for both SA (143,628/μL) and AA (45,649/μL) than in its presence: SA (32,400/μL) and AA (45,421/μL).

In diabetes as a pre‐existing condition (Figure 2C), the overall trend was similar to gout, where the mean synovial leucocyte counts in SA were lower in the absence (134 625/μL) than in the presence (159 186/μL), whereas for AA, the readings were comparable (presence: 41056/μL; absence: 47043/μL). The trend for immunosuppressed patients (Figure 2D) was similar to that of pseudogout, with a higher mean synovial leucocyte count for both SA and AA in the absence (SA: 145991/μL; AA: 46623/μL) in comparison to the presence (SA: 64800/μL; AA: 35603/μL) of immunosuppression.

### 3.3 Logistic Regression Analysis

The regression analysis correctly predicted (accuracy) the SA or AA in 323/360 (89.72%) patients in the entire cohort (*N* = 360). The sensitivity (correct predictions for SA) was 50% (28/56), while the specificity (correct predictions for AA) was 97.04% (295/304). In comparison, when a synovial leucocyte count threshold of ≥ 100,000/μL was used, the sensitivity was 48.21% (27/56) and specificity 88.49% (269/304). Similarly, a threshold of ≥ 50,000/μL yielded a sensitivity and specificity of 64.25% (36/56) and 69.74% (292/304), respectively. The confusion matrices with different measured statistical parameters are provided in Table 2.

**TABLE 2:** Summary of the parameters derived from confusion matrices from the logistic regression model and the two synovial leucocyte count thresholds of 100,000/μL and 50,000/μL.

Method	TP #	FP #	TN #	FN #	Sensitivity (%)	Specificity (%)	PPV (%)	NPV (%)	LR+ (%)	LR− (%)	Accuracy (%)
Logistic regression	28	28	295	9	50	97.04	75.68	91.33	16.89	0.52	89.72
≥ 100 000/μL	27	36	268	29	48.21	88.16	42.86	91.38	4.34	0.59	81.94
≥ 50 000/μL	36	92	212	20	64.29	69.74	28.13	91.38	2.12	0.56	68.89

Abbreviations: FN, false negative; FP, false positive; LR−, negative likelihood ratio; LR+, positive likelihood ratio; NPV, negative predictive value; PPV, positive predictive value; TN, true negative; TP, true positive.

The *χ* ^2^ value of the logistic regression model was estimated to be 123.82 with 26° of freedom and a *p* value of < 0.001, suggesting a zero probability of observing a *χ* ^2^ value of 123.82 if the null hypothesis was upheld. Furthermore, a *p* value of < 0.001 indicated a statistically significant relationship at the 5% level. The −2 Log‐Likelihood value was 187.38, which indicated the model's fit relative to a perfect fit. The Cox & Snell and Nagelkerke *R* ^2^ values of 0.29 and 0.5 meant that these models could explain 29% and 50% of the observed variances, respectively. McFadden's *R* ^2^ was estimated to be 0.4. These data collectively established that the model was significant.

**FIGURE 3:**
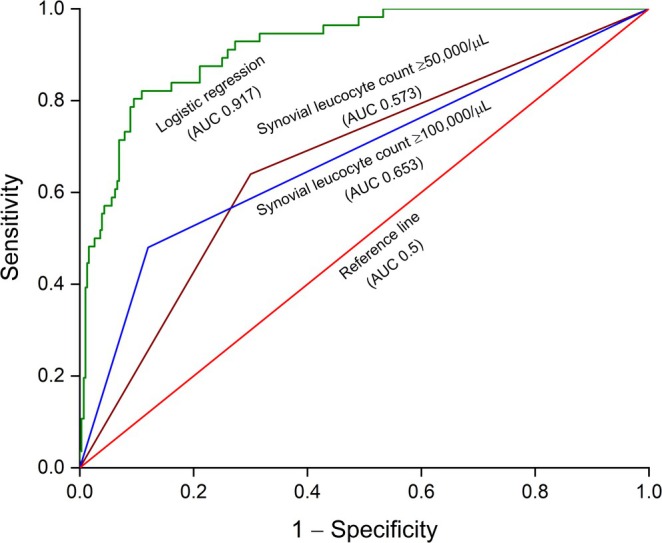
The ROC curve (green trace) using the binary logistic regression model used in this study (AUC = 0.917) showed improved modeling of the data compared to the synovial leucocyte‐based thresholds of ≥ 100,000/μL (blue trace) with an AUC of 0.653 and ≥ 50,000/μL (brown trace) with an AUC of 0.573. The reference line (AUC = 0.5) is marked in red.

The data from the logistic regression analysis, including the regression coefficients (*b*_*x*_) and *p* values, are shown in Table 3 and were used to develop an interactive model to determine the probability % of SA when the independent variables were adjusted. With a measured AUC of 0.917, the ROC curve (Figure 3) also demonstrated the logistic regression model's improved performance compared to the synovial leucocyte count‐based thresholds of ≥ 100,000/μL (AUC 0.653) and ≥ 50,000/μL (0.573).

**TABLE 3:** The obtained model after logistic regression analysis of the dataset. The numerical values were approximated to the second decimal place, while *p* values < 0.05 (statistical significance) were marked in bold fonts.

	Coefficient b_x_	Standard error	*Z*	*p*	Odds ratio	95% CI
Constant	−1.41	1.4	1	0.315	0.24	0.02–3.83
Gender—Male	0.35	0.43	0.82	0.412	1.42	0.61–3.29
Age	−0.05	0.02	3.1	**0.002**	0.95	0.92–0.98
Immunosuppression—Present	1.25	1.42	0.88	0.38	3.47	0.22–56.12
Diabetes—Present	0.92	0.61	1.5	0.134	2.5	0.75–8.28
Gout—Present	−1.71	0.77	2.23	**0.026**	0.18	0.04–0.81
Pseudogout—Present	1.93	1.43	1.35	0.177	6.91	0.42–114.22
Crystals—Yes	−2.07	0.54	3.84	**< 0.001**	0.13	0.04–0.36
Autoimmune arthritis—Present	−3.16	1.49	2.13	**0.033**	0.04	0.0–0.78
Single joint—No	−0.66	0.57	1.15	0.249	0.52	0.17–1.59
Aspirate cell count/μL	0	0	4.49	**< 0.001**	1	1–1
CCI 1	−0.64	0.88	0.73	0.466	0.53	0.09–2.94
CCI 2	1.25	0.83	1.52	0.129	3.51	0.69–17.73
CCI 3	2.33	0.97	2.41	**0.016**	10.32	1.54–68.97
CCI 4	1.23	1.28	0.96	0.335	3.42	0.28–41.75
CCI 5	1.36	1.09	1.25	0.21	3.91	0.46–32.88
CCI 6	1.8	1.27	1.41	0.157	6.02	0.5–72.58
CCI 7	1.1	1.5	0.73	0.465	3	0.16–56.85
CCI 8	−16.12	8107.4	0	0.998	0	0–∞
CCI 9	−19.08	16289.72	0	0.999	0	0–∞
CCI 10	−18.23	16289.72	0	0.999	0	0–∞
CCI 11	2.22	1.81	1.23	0.219	9.18	0.27–316.38
Joint aspirated—Shoulder	3.04	1.63	1.87	0.062	20.96	0.86–512.76
Joint aspirated—Wrist	−0.91	1.9	0.48	0.632	0.4	0.01–16.73
Joint aspirated—Hip	3.44	1.58	2.18	**0.029**	31.34	1.41–694.72
Joint aspirated—Knee	1.28	1.3	0.99	0.323	3.6	0.28–45.56
Joint aspirated—Ankle	0.4	1.5	0.27	0.79	1.49	0.08–28.46

Abbreviations: CCI, Charlson comorbidity index; CI, confidence interval.

## 4 Discussion

The logistic regression analysis highlighted the complex interplay between correlative factors that contributed to a diagnosis of SA and its distinction from AA. The male gender showed a positive correlation, although it was insignificant. Interestingly, age showed a significant correlation (*p* < 0.05) to SA characterized by a negative regression coefficient. Immunosuppression, on the other hand, despite an insignificant correlation, demonstrated a positive regression coefficient and an odds ratio of 3.47. The correlation to SA also varied among upper and lower limb joints. In the upper limb, the shoulder joint elicited a positive correlation, whereas the wrist joint demonstrated a negative correlation (both statistically insignificant). Conversely, all the lower limb joints (knee, hip, and ankle) exhibited positive regression coefficients. However, only the hip joint demonstrated a significant correlation (*p* < 0.05), a worthwhile finding from a clinical perspective.

The presence of comorbidities (e.g., diabetes, pseudogout) positively correlated with SA, indicating their contributory roles. Conversely, gout and autoimmune arthritis showed significant (*p* < 0.05) yet negative correlations. The presence of crystals in joint aspirates also exerted a significantly negative correlation, and it would be apt to view the results through the lens of coexisting gout or pseudogout. It is known that, especially in monoarthritic cases, crystal‐induced arthritis and SA often coexist [31], and the presence of crystals does not exclude SA or *vice versa* [32, 33]. In the presence of gout, the mean leucocyte count in SA was threefold higher than in AA. However, a similar trend emerged even without gout, making it difficult to comment on whether gout had an additional impact on synovial leucocyte count.

Further interesting patterns emerged when the mean synovial leucocyte counts were filtered by the presence or absence of diabetes and immunosuppression. In the presence of diabetes, the mean leucocyte count was higher in SA than in AA, and this trend was also observed in its absence. However, although a similar trend was noticed for immunosuppression, the mean synovial leucocyte count in SA without immunosuppression was higher than in those with it. However, the gross disparity between the number of SA (*N* = 56) and AA (*N* = 304) cases in the entire cohort makes it difficult to draw firm conclusions, and further verification from analysis on larger datasets with a comparable number of SA and AA cases is necessary. Unfortunately, avoiding such disparity of instances in a retrospective study like this one is difficult. Moreover, attempting to enforce a numerical equivalence, for example, by randomly selecting 56 AA cases to match the 56 SA ones, risks leaving useful variables unexplored and excluded from the model, thereby rendering it untrustworthy with poor performance.

The logistic regression modeling of SA, or its absence, outperformed diagnoses made on isolated and arbitrary thresholds, such as the synovial leucocyte counts of ≥ 100,000/μL or ≥ 50,000/μL. The improvement elicited by logistic regression, often remarkably, was grossly reflected in many of the considered parameters, including specificity and accuracy, and AUC in ROC curves, demonstrating its edge over synovial leucocyte count‐driven models. Regrettably, regarding sensitivity, the logistic regression model failed to demonstrate an edge over the synovial leucocyte count thresholds. Its sensitivity was less than the 50,000/μL threshold, although slightly better than 100,000/μL.

However, when estimated over diverse parameters, including accuracy and AUC ROC, the logistic regression model outperformed the 50,000/μL and 100,000/μL thresholds. Similarly, in terms of specificity, it outperformed the other two threshold‐driven models. Taken together, despite a disappointing show on sensitivity, which may stem from a mismatch between the numbers of SA and AA cases in the dataset, the overall performance of logistic regression made a valid case over the other two threshold‐based models. However, acquiring a trustworthy, robust dataset with sufficient case numbers and homogeneously distributed independent variables across genders is necessary to enhance the performance of such a machine learning platform.

As an online tool, DATAtab was a helpful and intuitive resource that provided step‐by‐step guidance and explanations that clinicians would find useful. The emergence of online platforms like DATAtab or other comparable software provides fresh opportunities for clinicians to explore their datasets with interactive visualization. Additionally, their integrated cloud computing systems obviate the need to download or run heavy applications and install dedicated workspaces with expensive data servers for saving data. This is particularly useful for improving the cost‐effectiveness of analysis, while it also aids data or skill transfer.

It is challenging to determine the optimal case number when building such a dataset. On realistic grounds, it may be implausible to collect data from a sufficient number of patients, especially in rare diseases. Additionally, even in an adequate dataset, further specifics, such as the optimal ratios of male‐to‐female patients or SA‐to‐AA episodes, need to be resolved. Perhaps no standard guideline or *fit‐for‐all* model can explain the variance in all the datasets. With the increasing digitization of patient records, such a model might require further optimization and refinements using available local, regional, or national epidemiological data on the disease under consideration.

Only a CCI index of 3 exhibited a significant (*p* < 0.05) correlation with SA, while the remaining indices (1, 2, 4–11) did not. However, an interesting aspect was that the lower CCIs (1–7) demonstrated positive regression coefficients of lower magnitude (except for CCI 1, which had a low‐magnitude negative coefficient), whereas the regression coefficients increased in magnitude (roughly around −20) for higher indices (8–11). Notably, higher comorbidities did not always increase the probability of SA in a patient.

Standardization of the independent variables is necessary for developing models that are applicable across the cross‐section of various healthcare systems. However, from the perspective of this study, such standardization was avoided as the original scale of the independent variables was deemed necessary for regression analysis. Moreover, for a complex disease like SA that may involve multiple organs and systems, it is difficult to pinpoint the specific details of such variables. Medical organizations, such as the Royal College of Pathologists of Australasia or its equivalents, need to come forward and, when necessary, collaborate to suggest protocols and harmonize the current clinical and diagnostic paradigms.

Finally, the synovial aspirate leucocyte count, the pivotal topic of discussion here, showed a significant correlation with SA, with a *p* value of < 0.001. Thus, as an independent variable, the joint aspirate leucocyte count demonstrated an undeniable correlation to SA [34, 35]. Perhaps that also provided some rationality behind using arbitrary synovial count thresholds for diagnosing SA, or at least where the roots of such a pervasive concept rest. However, when taken in its entirety, the logistic regression analysis unveiled the precarious nature of any univariate diagnostic criterion of synovial leucocyte count and cautioned against singling out individual threads to judge the tapestry of a complex and multilayered pathology like SA.

A major strength of this study was its relatively adequate dataset, which allowed logistic regression to have ample ground for analysis, enabling predictive modeling to identify and correlate factors other than synovial cell count in the diagnosis of SA. However, the dataset suffered from its retrospective nature and associated bias, the identification of variables after confirmation of diagnosis (rather than prospectively), a lack of information limiting data collection, and, at times, an absence of data emerging from a standardized collection of cultures (including peripheral blood cultures). In a way, it highlighted the need for having a robust, well‐thought‐out, and well‐formulated study design to be in place before data collection ensues. Such a structured study design is only possible through collaboration, dialog, and cross‐pollination of ideas between the clinicians and data scientists. Future studies should include this vital range of information in their datasets. Conducting multi‐center trials on larger datasets, and, most importantly, datasets collected based on standardized independent variables (if applicable), would be necessary to maximize the benefits of such a modeling‐based approach, including diagnostics, for complex diseases like SA.

## 5 Conclusion

A retrospective study of a patient‐derived dataset comprising 360 aspirated inflamed joint episodes (15.56% SA and 84.44% AA) was conducted with statistical analysis and logistic regression to identify the correlation matrix of SA. The cohort featured 71.11% male and 28.89% female patients. Additionally, data on a set of independent variables—such as age, comorbidity (immunosuppression, gout, pseudogout, diabetes, autoimmune arthritis), CCI indices, aspirated joint (knee, shoulder, hip, ankle, wrist, elbow), and synovial aspirate cell count—were collected. A logistic regression analysis revealed a complex syntax of mechanical principles that drove the pathogenesis of SA (or its absence thereof) where patient age, presence of gout, involvement of the hip joint, synovial aspirate leucocyte count, and the presence of crystals in aspirated fluid significantly (*p* < 0.05) contributed toward a diagnosis of SA, often with high odds ratios.

The regression model overall outperformed the popular and yet inadequate (due to a lack of strong scientific rationale) guiding Australasian protocol, where synovial leucocyte counts of ≥ 100,000/μL or ≥ 50,000/μL are prioritized for a diagnosis of SA or bacterial arthritis, including the determination of sensitivity and specificity. The results firmly established the grounds for a multivariate correlation of disease etiology which, in conjunction with a robust and scientifically justifiable diagnosis, is also likely to guide the management protocols, with its scope need not be confined to SA only, but also inclusive of other multisystemic disorders. Finally, the availability of user‐friendly machine learning platforms is providing a range of tools for application in clinical practice and building models that may facilitate and upgrade—if not rectify—the medical wisdom that, despite serving humanity over millennia, needs a rehash and, fortunately, remains receptive to such emerging techniques.

## Author Contributions

S.B.: conceptualization, data curation, formal analysis, visualization, and writing – original draft preparation. I.C.H.: conceptualization, data collection, data curation, visualization, and writing – review and editing. S.V.: conceptualization, data collection, methodology, software, visualization, and writing – review and editing. R.W.B: conceptualization, data collection, visualization, and writing – review and editing. S.K.: conceptualization, data collection, visualization, and writing – review and editing. All authors have read and approved the final manuscript.

## Conflicts of Interest

The authors declare no conflicts of interest.

## Supporting information


**Supporting Information S1:** Original dataset collated as an MS Excel spreadsheet.

## Data Availability

All data are incorporated into the article and its online Supporting Information. Further information and clarification(s) may be obtained from the corresponding author upon reasonable request.
